# Metabolism and the Mind: Investigating the Link Between Glucose Control and Reinforcement Learning in Humans

**DOI:** 10.1016/j.bpsgos.2025.100645

**Published:** 2025-10-29

**Authors:** Hugo Fleming, Martyna K. Stasiak, Isabel Lau, Annalise Whines, Sara Z. Mehrhof, Camilla L. Nord

**Affiliations:** a Medical Research Council Cognition and Brain Sciences Unit, University of Cambridge, Cambridge, United Kingdom; b Social, Genetic and Developmental Psychiatry Centre, King’s College London, London, United Kingdom; c Department of Experimental Psychology, University of Oxford, Oxford, United Kingdom; d Department of Physiology, Anatomy and Genetics, University of Oxford, Oxford, United Kingdom; e Department of Psychiatry, University of Cambridge, Cambridge, United Kingdom

**Keywords:** Computational psychiatry, Depression, Glucose tolerance, Interoception, Metabolic psychiatry, Reward learning

## Abstract

**Background:**

Signals from the body profoundly influence cognition. This process is known as interoception, and has been extensively studied in the cardiac, respiratory, and gastric domains; in contrast, metabolic influences remain poorly understood. Here, we focus on the link between glucose control and cognition, motivated by the observation that there is substantial, unexplained comorbidity between type 2 diabetes and depression. In rodents, insulin modulates dopamine signaling in the ventral striatum. We therefore hypothesized that, in humans, differences in glucose control would be associated with altered reward learning.

**Methods:**

To test this hypothesis, we recruited 48 participants from the general population, who each completed a glucose tolerance test, a monetary reward learning task known to relate to dopamine function, and mental health questionnaires. We fitted an established reinforcement learning model to the task data to obtain computational parameters characterizing participants’ learning, and then examined the associations between these parameters and their glucose control.

**Results:**

We discovered that poorer glucose control was associated with greater reliance on recent rewards during learning, which was in turn associated with higher levels of depression symptoms. There was also more modest evidence for the association between glucose control and depression symptoms.

**Conclusions:**

Together, our results identify a specific neurocognitive process, reward learning, by which metabolic information may influence cognition, and which may explain the link between metabolic diseases such as type 2 diabetes and depression.

## Plain Language Summary

Signals from the body influence the way we think and feel. We studied whether differences in blood sugar regulation affect learning and mood. A total of 48 adults completed tests measuring blood sugar control, reward-based learning, and mental health. Those with poorer blood sugar control relied more on recent rewards when learning—a pattern linked to higher depression symptoms. Although preliminary, these results suggest that metabolic signals may impact reward learning beyond eating behavior, connecting metabolism and mental health. This could help explain why type 2 diabetes and depression often occur together.

Neuroscientists have studied the ways in which physiological signals are processed by the brain since Sherrington (1), who first coined the term interoception. Since then, the field has grown substantially, and researchers have identified a range of bodily signals, including heart rate, breathing, and core temperature, that influence cognition and emotion (2,3). Individual differences in interoception have also been linked to several common mental health conditions, including anxiety, depression, and psychosis (4). Understanding how bodily signals shape cognition is therefore critical not only for elucidating normal brain function, but also for uncovering the mechanisms underlying psychiatric illness.

One domain of bodily signals that remains somewhat overlooked, however, is that of metabolic information. The brain plays a central role in energy regulation: it must monitor energy availability and, weighing the benefits of pursuing potentially rewarding activities against their anticipated energetic costs (5,6), adjust behavior and cognition accordingly. But how does the brain achieve this, and how does this metabolic interoception shape decision making? Existing human studies on metabolism and cognition have largely focused on eating behavior and food rewards [e.g., (7, 8, 9)]. However, the high comorbidity between metabolic disorders and depression (10,11) indicates that metabolic signals may influence decision making more broadly. In particular, it has been suggested that the neural systems regulating energy balance overlap with those that control reward learning and motivation (12, 13, 14). In the current study, we set out to test whether metabolic signals have domain-general effects on cognition, i.e., whether they shape decisions even in tasks unrelated to food or eating.

Evidence from rodent experiments strongly supports this possibility. Insulin, a key metabolic hormone, readily crosses the blood-brain barrier (15), and insulin receptors are densely expressed on dopaminergic neurons in the ventral striatum, a region critical for reward learning and motivation (16). Blocking insulin signaling in this area disrupts flavor and place preference learning (17), while brainwide insulin receptor knockout induces depression-like behavior on the tail suspension and forced swim tests (18). These studies illustrate what happens when brain insulin signaling is impaired, which is an important aspect of human type 2 diabetes. While this condition is primarily characterized by reduced peripheral insulin sensitivity, which affects blood glucose regulation, this develops in tandem with reduced neuronal insulin sensitivity (19). Together, these results suggest that insulin signaling may be an important link between metabolic and cognitive function, influencing variation in blood glucose control, reward learning, and affective symptoms.

Disrupted reward learning is a well-established feature of several mental health disorders. In humans, this is often studied within the framework of computational psychiatry, which has shown that impairments in reward processing are associated with depression symptoms, particularly anhedonia (20, 21, 22). The precise computational mechanisms are debated, with some studies implicating altered reward learning rates (23, 24, 25, 26), while others have emphasized reduced reward sensitivity (27, 28, 29). Given the heterogeneity of depression, it is likely that multiple pathways—each with distinct computational signatures—contribute to these effects (30). To characterize these mechanisms, we used an established paradigm, the probabilistic selection task, and fitted well-validated reinforcement learning models (31, 32, 33).

To assess glucose control, we used an oral glucose tolerance test (OGTT), in which participants consume a concentrated glucose solution and their glucose level is monitored as it rises and returns to baseline. A higher and more prolonged glucose spike reflects poorer glucose control, which is a marker for peripheral insulin resistance and elevated risk of type 2 diabetes (34). To enhance the OGTT, we used a continuous glucose monitor (CGM), a relatively new technology that provides glucose estimates every 2 minutes, in contrast to the 15-minute resolution of traditional finger prick testing (35).

The primary aim of this study was to investigate the relationship between glucose control and reward learning. Specifically, we hypothesized that participants with poorer glucose tolerance, as indexed by incremental area under the curve (iAUC), would show lower test-phase accuracy and altered learning parameters in computational models. Evidence for this relationship would suggest that metabolic signals shape decision making in a domain-general way, with potential implications for understanding comorbidity between metabolic and psychiatric disorders.

## Methods and Materials

### Preregistration

This study was preregistered on the Open Science Foundation (https://doi.org/10.17605/OSF.IO/UQ58Z); there were no deviations from this protocol. The task code, raw data, and analysis scripts are stored at https://osf.io/b9z5v/.

### Procedure

**Figure 1:**
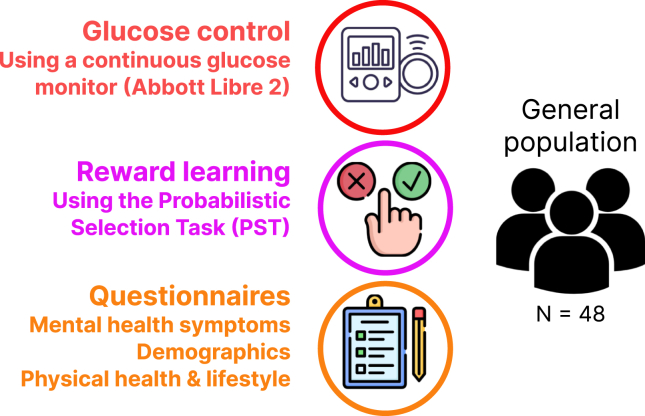
The study design. First, we administered an oral glucose tolerance test, quantifying participants’ ability to regulate their blood sugar levels. Next, they completed a reward learning task (to which we were subsequently able to fit a computational model and extract parameters representing their learning rates and decision noise). Finally, participants completed a set of mental and physical health questionnaires.

Participants attended the laboratory in the morning following an overnight fast (i.e., 7–14 hours without food or drinks except water, and no exercise or nicotine). A CGM was attached to the back of the left arm and allowed to calibrate for 1 hour. Then we began the OGTT: participants consumed 50 g of glucose dissolved in 250 mL of water, and their blood glucose was monitored over the next 2 hours. In this time, participants completed a reward learning task on the computer followed by several mental health questionnaires. Any remaining time could be spent sitting quietly (e.g., reading or working on a laptop). The study concluded at the end of the 2-hour monitoring window. This procedure is summarized in Figure 1.

### Participants

We recruited 56 participants from the general population around Cambridge. Eligible participants were ages 18 to 60 years, fluent in English, and not taking medications known to affect glucose regulation (e.g., insulin, steroids). We did not otherwise exclude individuals with mental or physical health conditions.

Following preregistered exclusion criteria or technical issues, 8 participants were excluded: 4 failed attention checks during the questionnaires and 4 were excluded because of connectivity issues with the CGMs. This left 48 participants in the final sample, exceeding our preregistered minimum of 46 (calculated to detect an effect size of *r* = 0.4 with 80% power at α = 0.05). Given the absence of prior studies linking glucose iAUC to cognitive performance, this moderate effect size (36) was chosen as a pragmatic benchmark, balancing statistical sensitivity with practical feasibility. It is consistent with individual differences research using a comparable probabilistic reward learning paradigm, where associations between reward learning and continuous traits (e.g., depression symptoms) typically fall in the range of *r* = 0.3 to 0.5 [e.g., (24,26)]. The study was approved by the Cambridge Psychology Research Ethics Committee (PRE.2022.078), and all participants provided written informed consent. Summary characteristics are shown in Table 1, with further detail provided in Table S1.

**Table 1:** Demographic Characteristics of the Sample

Characteristic	Mean (SD) [range] or *n*
Sample Size	48
Age, Years	41.2 (14.6) [18–60]
Sex	
Female	36
Male	12
Body Mass Index, by Group	
<18.5	2
18.5–24.9	26
25.0–29.9	8
≥30	12
Body Mass Index	25.6 (5.69) [17.6–39.3]

### Measures

#### Oral Glucose Tolerance Test

We followed the method described by Suez *et al.* (37) for using CGMs in the OGTT. A Freestyle Libre 2 sensor (Abbott Laboratories) was applied to the back of the participant’s left arm and allowed to calibrate for 1 hour. Participants then consumed 50 g of glucose dissolved in 250 mL of water (within 2 minutes). Glucose levels were tracked for 2 hours after ingestion.

The main outcome of interest was the iAUC, which comprises the area below the curve but above the fasting concentration of glucose. This metric, commonly used in human research (37,38), was preferred over total or net AUC because it accounts for individual differences in fasting glucose.

We computed iAUC using the trapezoid rule: for each pair of glucose readings, y_1_, y_2_, at time points t_1_, t_2_, $\text{area}=\frac{\left(y_{1}+y_{2}\right)}{2}\times \left(t_{2}-t_{1}\right)$. If either reading was below baseline, we used linear interpolation to calculate only the positive area; if both were below baseline, the interval was excluded. Finally, the incremental areas were summed to give the overall iAUC, with a higher iAUC indicating poorer glucose control.

We also report some validation analyses using fasting glucose, which was the initial CGM reading taken prior to participants consuming the glucose drink (i.e., after the overnight fast).

CGMs have been validated for people without diabetes (39,40), and recent studies have reported good agreement between CGMs and venous sampling iAUCs (*r* = 0.73) (41), with mean bias <5% (42), supporting their use in cognitive neuroscience studies.

#### Reward Learning Task

To assess reward learning, we used the probabilistic selection task (PST) (32,33), incorporating the affect ratings from Dercon *et al.* (31). A schematic of the task is provided in Figure S1. Participants chose between pairs of Japanese Hiragana symbols, each of which was associated with a different reward probability (20%, 30%, 40%, 60%, 70%, or 80%). Symbol-outcome mappings were randomized.

After instructions (including a mandatory comprehension check), participants completed a training phase comprising 6 blocks of 60 trials. Symbols were presented in fixed pairs (80%–20%, 70%–30%, 60%–40%). Feedback followed each choice: “Correct! You won 25 points”, “Incorrect”, or “No response detected”. Participants also rated their momentary happiness, confidence, or engagement on that trial using a sliding scale. At the end of each block, they also rated their fatigue.

In the test phase (60 trials), all symbol pairings were shown twice. No feedback was given, and participants were instructed to choose the symbol that “felt most correct” on each trial.

#### Mental and Physical Health Questionnaires

We administered 7 self-report symptom scales assessing depression (Patient Health Questionnaire [PHQ-9]) (43), anxiety (Beck Anxiety Inventory [BAI] and Generalized Anxiety Disorder-7 scale) (44,45), anhedonia (Snaith-Hamilton Pleasure Scale [SHAPS]) (46), apathy (Apathy Evaluation Scale [AES]) (47), impulsiveness (Impulsive Behavior Short Scale-8 [I-8]) (48), and physical activity (the General Practice Physical Activity Questionnaire) (49). The BAI contained an “easy” attention check (Press “not at all”), while the I-8 and AES each included a “hard” check (do they “work forty hours a day” and have they “used a computer”). Participants were excluded if they failed the easy check or both hard checks. We also asked additional questions about lifestyle and physiological variables, including smoking status, sleep quality, and (for female participants) menstrual phase. At the end of the questionnaires, participants confirmed whether they had done the overnight fast (with reassurance that their payment would be unaffected). All participants responded “yes.”

### Preregistered Analyses

Our preregistered primary hypothesis was that participants’ glucose control would be associated with reward learning. We tested this both model-agnostically (by correlating glucose iAUC with accuracy on the PST) and using computational modeling. For the latter, we fitted a well-established hierarchical reinforcement learning model (33) using Hamiltonian Markov chain Monte Carlo (MCMC) in *Stan* (50).

On each trial, the probability of choosing symbol i over j was modeled as a logistic function of the difference in their expected values (Q values), scaled by the participant’s outcome sensitivity (β).

(1) $$p_{choose\_i}=logistic\left(\beta \times \left(Q_{i}-Q_{j}\right)\right)$$

Following each choice, the Q value of the chosen option was updated via a Rescorla-Wagner rule:

(2) Q(t+1)=Q(t)+α(R(t)−Q(t))

We tested 2 model variants: one with a single learning rate (α) and one with separate learning rates for positive and negative prediction errors (reward and loss learning rates). In previous studies, the reward learning rate parameter been linked to dopamine function (32,33).

Note that we did not include separate reward and punishment sensitivities as had been anticipated in our preregistration because this led to model nonidentifiability due to a trade-off with the corresponding learning rates.

Both models fitted well (no divergences, split-Rhat < 1.1, energy Bayesian fraction of missing information > 0.3, maximum treedepth < 10) (51), and posterior predictions visually matched the empirical data. Parameter recovery analyses indicated very good correlations between observed and recovered parameters (*r* = 0.94, 0.95, and 0.90 for reward learning rate, loss learning rate, and outcome sensitivity parameters, respectively), validating our use of these models in our analysis here (see Figure S3).

Approximate leave-one-out cross-validation strongly favored the 2 learning rate model, which we used in all subsequent analyses (details of fitting, priors, and model comparison are provided in the Supplement). We extracted individual estimates of reward learning rates and correlated these with glucose iAUC. As MCMC yields posterior distributions rather than point estimates, we computed correlations across all (4000) posterior draws, giving posterior distributions for each correlation. We report both the posterior mean and the 90% highest density interval (HDI), a commonly used interval in Bayesian analysis that balances sensitivity and interpretability (52). Correlation strength is interpreted using standard thresholds: ∼0.1 (small), ∼0.3 (moderate), and ∼0.5 (large) (36).

Other exploratory analyses (associations between glucose iAUC and questionnaire measures) were conducted using Bayesian regressions implemented in *rstanarm* (53).

### CGM Validation Dataset and Analyses

Although CGMs are well validated for tracking within-subjects fluctuations, their use for measuring between-subjects differences is newer. To assess its validity, we ran confirmatory regressions to test whether fasting glucose and iAUC were associated with age and adiposity. To increase power, we combined data from this study (*N* = 48) with data from a later study using the same OGTT protocol (*n* = 52; https://osf.io/rcusg/), yielding a combined validation sample of *n* = 100.

## Results

### Validation Analyses

**Figure 2:**
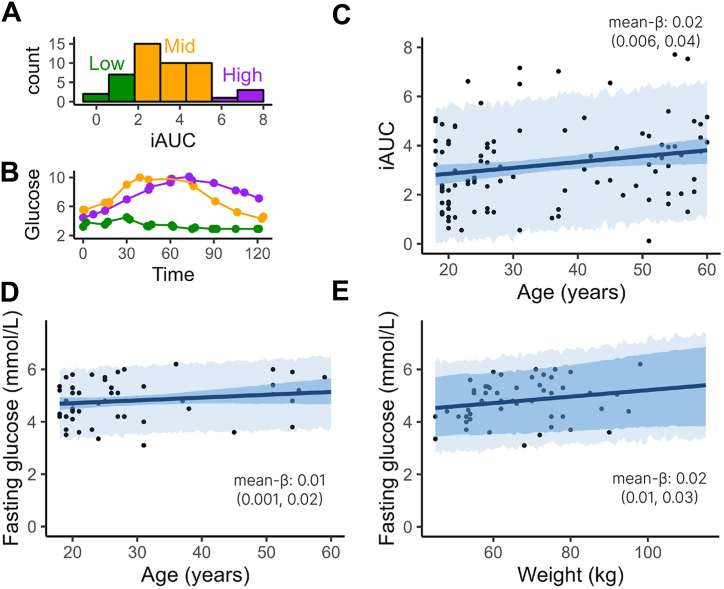
Continuous glucose monitor parameters and their associations with known metabolic risk factors. **(A)** Histogram of glucose incremental area under the curve (iAUC) values. **(B)** Glucose dynamics for 3 example participants with low, medium, and high iAUCs. **(C–E)** Results of the validation analyses. All plotted relationships show regression terms whose 90% highest density interval (HDI) did not cross zero, indicating credible effects. Panels show the relationships **(C)** between age and glucose iAUC, **(D)** between age and fasting glucose, and **(E)** between adiposity (weight adjusted for height) and fasting glucose. Plots show the posterior mean regression line (solid), the 90% HDI for the regression line (dark shading), and the 90% HDI for predicted observations (light shading). Empirical data are plotted in black for reference.

As CGMs are a relatively new method in cognitive neuroscience, we first validated their use by examining associations between key glucose metrics (iAUC and fasting glucose) and established predictors of glucose tolerance (age and adiposity). We replicated these analyses in a larger, combined dataset that included CGM data from a subsequent study (see the Methods and Materials for details). The results, plotted in Figure 2, showed that iAUC was positively associated with age in both our primary dataset (mean β = 0.03; 90% HDI, 0.007 to 0.06) and in the combined dataset (mean β = 0.02; 90% HDI, 0.006 to 0.04). Fasting glucose was also associated with age in the combined dataset (mean β = 0.01; 90% HDI, 0.001 to 0.02), although this effect was not significant in the smaller sample (mean β = 0.01; 90% HDI, −0.005 to 0.03).

iAUC showed no consistent association with adiposity (measured as the effect of weight after controlling for height: mean β = 0.01, 90% HDI, 0.01 to 0.04; mean β = −0.002, 90% HDI, −0.02 to 0.01), but fasting glucose concentration was associated with adiposity in both datasets (mean β = 0.02, 90% HDI, 0.005 to 0.03; mean β = 0.02, 90% HDI, 0.01 to 0.03).

Taken together, these results suggest that our preregistered primary outcome, glucose iAUC, may not fully capture glucose tolerance. Fasting glucose showed more consistent associations with known metabolic risk factors and may offer a more stable indicator. While iAUC remains valuable as a measure of glucose dynamics, it may benefit from being complemented by more stable measures such as fasting glucose. We therefore include fasting glucose as a sensitivity analysis in our subsequent results, where it successfully replicates our key findings.

### Primary Analysis: Glucose Control Correlates With Reward Learning

**Figure 3:**
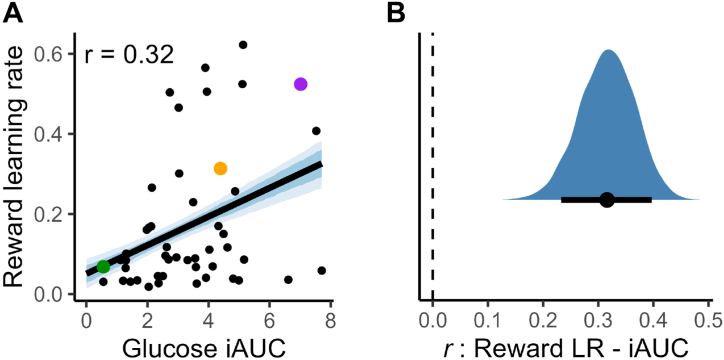
Graphs of the correlation between glucose incremental area under the curve (iAUC) and reward learning rate (LR). Participants with higher iAUC (indicating worse glucose tolerance) tended to have higher reward LRs. Panel **(A)** plots the relationship as a regression (black line, with the 66% and 90% highest density intervals [HDIs] in blue shading). The example participants with low/medium/high iAUC, identified in Figure 1, are highlighted here in green, orange, and purple, respectively. Panel **(B)** shows the full posterior distribution of the correlation in blue, with the mean and 90% HDI plotted in black beneath.

As preregistered, we tested the hypothesis that glucose control would be associated with reward learning. Using a validated reinforcement learning model (31), we estimated each participant’s reward learning rate and correlated it with their glucose iAUC. This correlation was significant (posterior mean *r* = 0.32; 90% HDI, 0.23 to 0.40), indicating that participants with poorer glucose control tended to update their behavior more rapidly following rewards (Figure 3). The full posterior distribution was above zero, providing strong evidence for a positive correlation.

A similar association was found for the loss learning rate (mean *r* = 0.23; 90% HDI, 0.16 to 0.31), whereas outcome sensitivity was not reliably linked to iAUC (66% HDI overlapped zero).

Given the limitations of iAUC as a stand-alone measure (see above), we repeated these analyses using fasting glucose. The results were virtually identical: both reward and loss learning rates were positively correlated with fasting glucose (mean *r* = 0.31, 90% HDI, 0.22 to 0.40; and mean *r* = 0.31, 90% HDI, 0.21 to 0.41, respectively). We found no evidence that glucose iAUC predicted overall task accuracy (in either training or test phases; 66% HDIs overlapped 0), suggesting that glucose control relates not to overall performance but rather to how participants update their choices. To explore this, we tested for a relationship between glucose iAUC and win-stay behavior. We observed modest evidence for such a link (mean *r* = 0.14; 90% HDI, −0.10 to 0.38); although the interval overlapped 0, the posterior probability that the correlation was positive was nevertheless 85%, which still partially supports an effect.

### Exploratory Analyses

**Figure 4:**
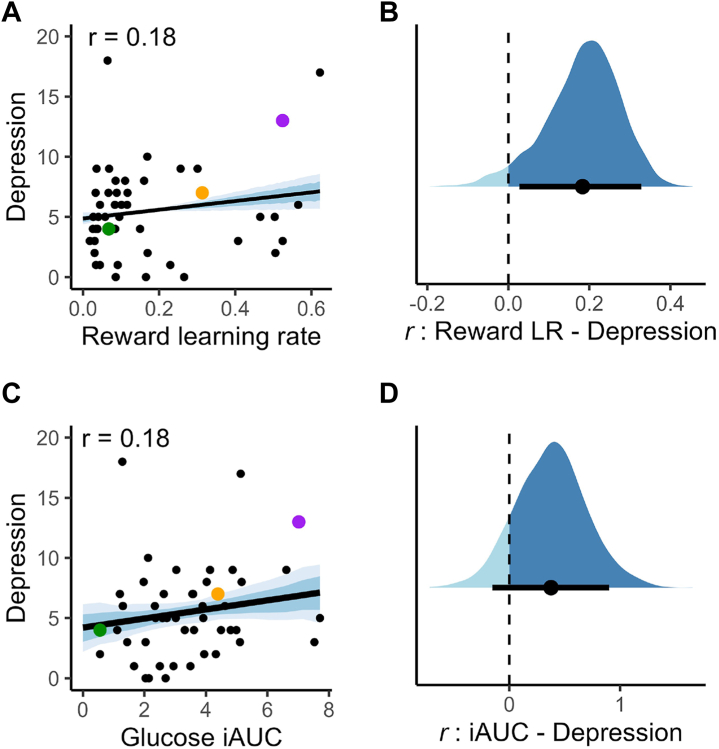
Correlations of depression symptoms with reward learning rate (LR) and glucose incremental area under the curve (iAUC). **(A, B)** Higher reward LR was associated with worse depression symptoms (Patient Health Questionnaire-9 score). **(C, D)** There was also a weaker, though generally positive, relationship between depression symptoms and glucose iAUC (note that the 90% highest density interval [HDI] for this effect crossed zero—see discussion in main text). Panels **(A)** and **(C)** plot these relationships as regressions (black line, with the 66% and 90% HDIs in blue shading). The example participants with low/medium/high iAUC, identified in Figure 1, are highlighted here in green, orange, and purple, respectively. Panels **(B)** and **(D)** display the full posterior distributions in blue, with the means and 90% HDIs plotted in black beneath.

We also explored the associations between model parameters, glucose control, and mental health symptoms. Reward learning rate showed small positive associations with several symptom measures: depression (PHQ-9 score: mean *r* = 0.18, 90% HDI, 0.03 to 0.33) (Figure 4A, B), anhedonia (SHAPS score; mean *r* = 0.19, 90% HDI, 0.05 to 0.33), and apathy (AES score; mean *r* = 0.19, 90% HDI, 0.06 to 0.32).

Glucose control showed a weaker and less conclusive link to depression symptoms (*r* = 0.18, 90% HDI, −0.06 to 0.42) (Figure 4C, D), but the posterior probability of a positive correlation was still relatively high (89%), consistent with a possible association (52). No reliable associations were found between glucose control and other mental health measures (66% HDIs for anxiety, anhedonia, apathy, and impulsiveness all overlapped 0).

### Changes in Affect Ratings

Participants’ subjective affect ratings declined slightly over the course of the training phase. Using Bayesian mixed-effects regression, we estimated that happiness ratings started at 57.6% on the first trial and subsequently declined by 0.08 percentage points per trial (90% HDI, 0.04 to 0.12); confidence ratings started at 37.8% and declined by 0.11 percentage points per trial (90% HDI, 0.06 to 0.16), and engagement ratings started at 65.9% and declined by 0.21 percentage points per trial (90% HDI, 0.14 to 0.27).

We also examined whether these declines were modulated by glucose control or learning rate. Higher reward learning rates were associated with a slower decline in engagement over time (interaction β = 0.42, 90% HDI, 0.05 to 0.80). No other significant effects were found.

## Discussion

In a general population sample (*N* = 48), poorer glucose control was associated with greater reliance on recent rewards during decision making, which in turn predicted higher depression scores. While the findings are correlational and do not establish causality, they do point to a specific neurocognitive profile linked with metabolic ill-health: individuals with higher learning rates tended to have worse glucose control.

Compared with other interoceptive domains, much less is known about how metabolic information shapes cognition. Most prior research has focused primarily on insulin’s role in eating behavior [e.g., (54)]. Here, we show that there is a broader interaction with decision-making processes. The link between glucose tolerance and increased reward learning rates suggests variation in the way participants weigh recent versus past outcomes, rather than a simple difference in the level of performance (an interpretation supported by our model-agnostic analysis). This pattern is consistent with theories proposing a role for metabolic state in modulating reinforcement learning [e.g., (12)].

Individual differences in learning rates may reflect variation in dopaminergic signaling (32,33), which is in turn modulated by central insulin. In animals, striatal insulin receptor sensitivity alters dopamine release and reward learning (17,18). In humans, intranasal insulin affects resting-state activity in dopaminergic regions including the striatum and prefrontal cortex (55, 56, 57), and may offer a tool for probing causal effects on reward learning (58,59). Future work could combine such interventions with computational modeling to test these mechanisms more directly.

Our results also point to new opportunities for mental health research. Depression and type 2 diabetes are highly comorbid, a fact which has led some authors to speculate that they may share underlying mechanisms (11,15,60). Based on our results, one possibility is that central insulin resistance causes changes in reward learning, which then contributes to the reward and motivational deficits seen in depression. If so, then improving insulin sensitivity could be an effective means of reducing depression symptoms. This suggestion is supported by several findings from the literature. For example, Hanssen *et al.* (61) found that an acute dose of the GLP-1 agonist liraglutide (which enhances peripheral insulin secretion, as well as having direct effects on brain reward pathways) led to improved motivation for participants with lower baseline insulin sensitivity. This converges with preliminary evidence from randomized controlled trials that GLP-1 agonists reduce depression symptoms (62).

A separate line of research has shown that both exercise (63, 64, 65) and dietary interventions (66, 67, 68) can have significant antidepressant effects. In both cases, the underlying mechanism remains poorly understood; however, one compelling explanation, supported by our results here, is that improved global insulin sensitivity mediates their antidepressant effects [e.g., see discussion in (69)].

Interestingly, our findings differ from those of Hanssen *et al.* (70), who reported lower learning rates in individuals with reduced peripheral insulin sensitivity. However, their task focused on sensory rather than reward learning and used a different computational model (the hierarchical Gaussian filter vs. Q-learning in the current study). More importantly, Hanssen *et al.* (70) studied selected groups (lean and obese), while we sampled from the general population without selecting for metabolic health. Our transdiagnostic approach aimed to maximize generalizability (71), but likely included fewer individuals with severe insulin resistance. This may be critical, as previous work has identified an inverted-U relationship between adiposity and brain reward responses (72), and similar nonlinearities may exist between insulin sensitivity and learning. It is also worth noting that insulin transport across the blood-brain barrier is saturable (73), meaning that increases in peripheral insulin do not translate linearly to central nervous system insulin levels. This may contribute to individual differences in learning effects and could underlie nonlinear relationships between peripheral insulin sensitivity and reward processing. These complex relationships highlight the need for further studies using unified tasks and models across a wide range of metabolic profiles.

Some limitations of the current study should be acknowledged. First, while the OGTT offers a practical and widely used index of glucose control, it does not directly assess insulin sensitivity. Gold-standard alternatives such as the hyperinsulinemic-euglycemic clamp or the homeostatic model assessment for insulin resistance (74) require venous sampling and are less suitable for a cognitive neuroscience setting, in particular due to safety considerations related to inducing hyperinsulinemia. Given these constraints, we selected the OGTT as the most feasible approach, although this necessarily limits our ability to draw specific inferences about insulin resistance; therefore, we focus here on the measurement of glucose control instead. Similarly, metabolic regulation is influenced by a range of other physiological systems (e.g., hormonal axes such as cortisol or ghrelin, gut microbiota, and stress pathways) that were not measured here. Future work could incorporate such indices to better characterize the multifactorial basis of metabolic-cognitive interactions.

Second, our glucose control measures have technical limitations. Traditionally, the OGTT uses a fixed glucose dose, which may not scale proportionally with body size (75), and CGMs can yield noisier data within the first 24 hours due to local inflammatory effects (76,77). Nevertheless, our replication of key findings using fasting glucose mitigates these concerns and illustrates the value of including complementary metabolic measures.

Third, our sample was mainly female (36:12 female:male), and the average body mass index (25.6) was slightly lower than that of the UK general population [27.6; (78)], both of which may affect generalizability. Future studies could adopt a systematic sampling strategy to achieve gender balance or even oversample particular metabolic phenotypes (e.g., individuals with obesity or insulin resistance) to improve both representativeness and interpretability.

Finally, while we have highlighted one possible pathway—from impaired glucose control to altered learning and mood—the relationship is likely bidirectional (79), raising the possibility of positive feedback loops in which neurocognitive and metabolic processes reinforce one another. The current study was correlational and cannot support directional conclusions. Therefore, clarifying the causal structure of these associations will be an important goal for future work.

### Conclusions

We have identified an association between glucose control, reward learning, and depression symptoms. While these results are correlational and preliminary, they offer initial evidence that metabolic signals may influence reward processing beyond food-related behavior, potentially shaping broader processes of learning and mood. Given the complexity of metabolic regulation and the limitations of our sample and design, further research will be essential to clarify the directionality, mechanisms, and generalizability of these effects. Nevertheless, these findings highlight an underexplored relationship between metabolic and cognitive processes, with implications for understanding and treating comorbid mental and metabolic disorders.

## Supplementary Material

Supplemental Methods, Results, Figures S1–S4, Tables S1–S2

Key Resources Table
