# Supplementary material for: Metabolism and the Mind: Investigating the Link Between Glucose Control and Reinforcement Learning in Humans
Source: Biol Psychiatry Glob Open Sci. 2025 Oct 29;6(2):100645. doi: 10.1016/j.bpsgos.2025.100645 (PMC12768912; doi:10.1016/j.bpsgos.2025.100645)
Supplement: Supplemental Methods, Results, Figures S1–S4, Tables S1–S2 [file mmc1.pdf]

SUPPLEMENTARY INFORMATION

Metabolism and the Mind: Investigating the Link Between Glucose Control and Reinforcement Learning in Humans

Fleming *et al.*

Supplement

Probabilistic Selection Task Schematic

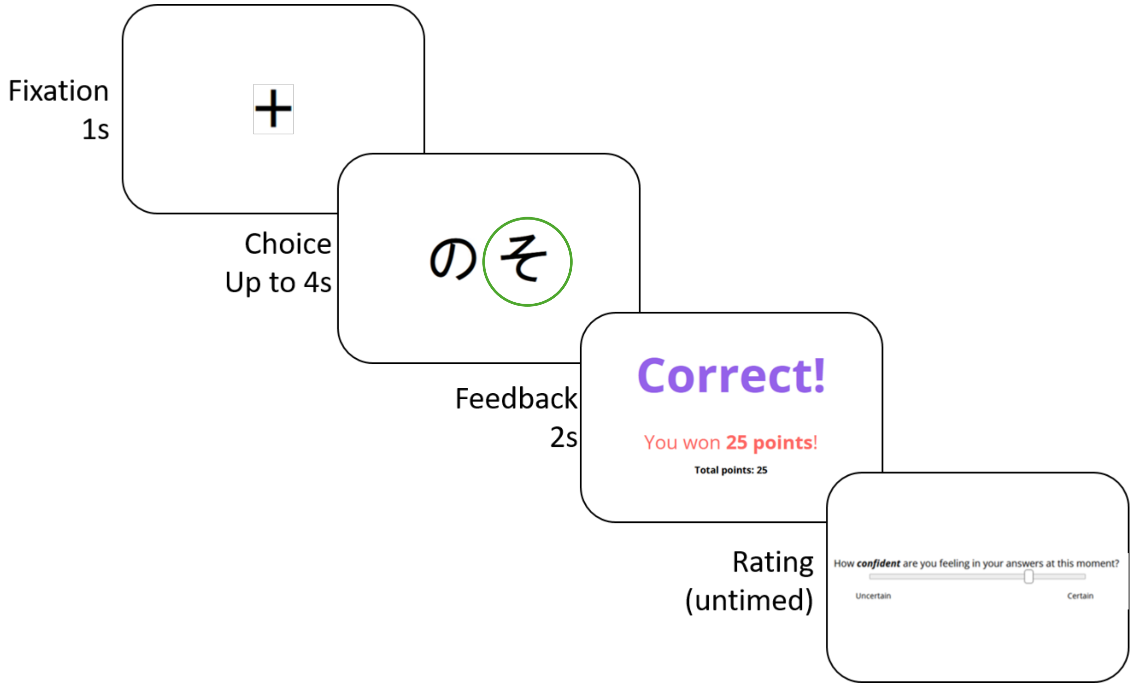

Figure S1. Schematic of the Probabilistic Selection Task. After an initial fixation period, participants were shown a pair of symbols (drawn from a set of six), and had to choose one by pressing the left or right arrow key. These symbols were associated with 20%, 30%, 40%, 60%, 70% or 80% chance of reward. In the training phase participants were then shown a feedback screen indicating whether they had won the reward (+25 points) or not. During the final test phase this screen was omitted. Finally, participants were asked a question about their current affective state.

Computational modelling: Further details

Full model specification

The full implementation of the model is provided at the OSF repository for this study, https://osf.io/b9z5v/. Below we set out the essential mathematical equations:

Learning

During the training phase, the values of the options (denoted by Q) were modelled using a Rescorla-Wagner learning rule, which augmented the value in Q by the prediction error weighted by the participant's learning rate, α:

$$Q_i(t) = Q_i(t-1) + \alpha\big(R - Q_i(t-1)\big)$$

In the two learning rate model, two different learning rates were applied, depending on whether a reward or loss was experienced on each trial.

After the training phase, during the test phase, there was no more feedback and so the Q values were no longer updated and were held constant for the remaining test trials.

Action selection

The response on each trial (choose stimulus *i*, choose stimulus *j*, or make no response) was modelled as being drawn from a categorical distribution:

$$response \sim categorical\begin{pmatrix} p_i \times (1 - p_{ignore}) \\ p_j \times (1 - p_{ignore}) \\ p_{ignore} \end{pmatrix}$$

Where p_i is the probability of choosing stimulus *i* over *j,* and is given by the difference between the Q-values of options i and j, multiplied by the sensitivity β and then passed through a logistic function. The complement p_j was simply $1 - p_i$.

$$p_i = logistic\left(\beta \times (Q_i - Q_j)\right)$$

The (few) trials where participants made no response at all were modelled by the parameter p_{ignore}.

Link functions

Both the learning rate and 'ignore' parameters were passed through logistic functions to ensure they fitted within the range 0-1, prior to being used in the learning/action stages of the model.

Priors

The model was specified hierarchically, i.e. the participant-level parameters (indexed 1…N) were themselves drawn from population-level distributions, as set out below.

$$\alpha_n \sim normal(\alpha_\mu, \alpha_\sigma)$$
$$\alpha_\mu \sim normal(0,1)$$
$$\alpha_\sigma \sim exponential(1)$$

$$\beta_n \sim normal(\beta_\mu, \beta_\sigma)$$
$$\beta_\mu \sim normal(0,2)$$
$$\beta_\sigma \sim exponential(1)$$

$$ignore_n \sim normal(ignore_\mu, ignore_\sigma)$$
$$ignore_\mu \sim normal(-2,1)$$
$$ignore_\sigma \sim exponential(1)$$

Model fitting and comparison

We fitted two versions of this computational model, one which contained just a single learning rate for each participant (as written out above; termed the Base model) and another which used two learning rates, one for reward trials and another for losses (referred to as the 'Base + 2LR' model). Both models were coded in Stan and fitted using Hamiltonian MCMC, with 4 chains for 1000 warmup and 1000 sample iterations. Both models fitted well (no divergences, split-Rhat < 1.1, E-BFMI > 0.3, maximum treedepth < 10; [1]), and posterior predictions visually matched the empirical data.

We compared their relative performance using approximate leave-one-out cross-validation (2). As shown in Figure S2 below, this substantially favoured the Base + 2LR model, which was ahead by approximately 16 standardised units of distance. Therefore this model was used for all subsequent analyses.

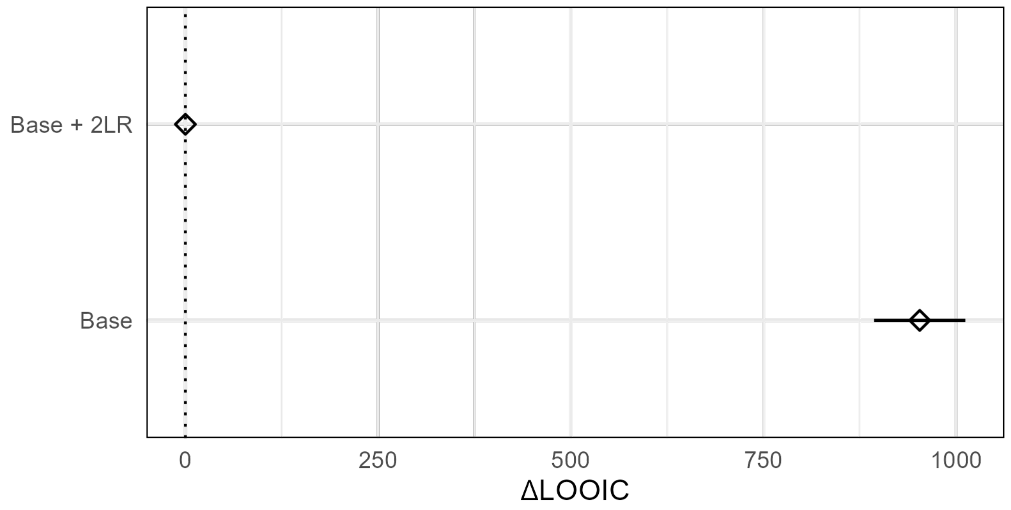

Figure S2. Model comparison showing substantially better approximate leave-one-out cross-validation performance for the model with two learning rates (Base + 2LR) compared with the single learning rate (Base) model.

Parameter recovery analyses

Parameter recovery was assessed by simulating new data from the empirical parameter estimates for each participant, refitting the model to these simulated datasets and comparing the recovered parameters to the originals. Recovery was very good: for reward learning rate, loss learning rate and outcome sensitivity, the correlations between empirical and recovered parameters were r = 0.94, 0.95 and 0.90 respectively (Figure S3).

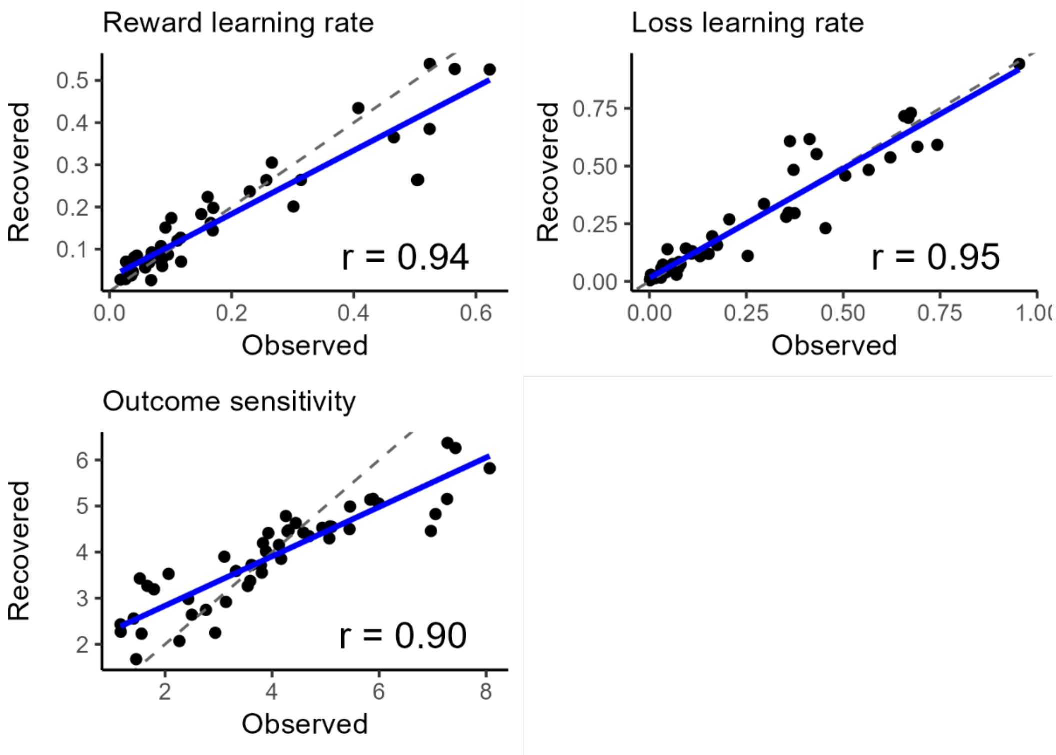

Figure S3. Parameter recovery results. All three parameters showed good recovery, with correlations of *r* = 0.94, 0.95 and 0.90 for reward learning rate, loss learning rate and outcome sensitivity respectively. Dashed grey line indicates perfect recovery (y = x), while the solid blue line shows the calculated regression line.

Supplementary results

To validate the reward learning task, we checked whether people's behaviour was consistent with previous studies. As shown in Figure S4A, all participants gradually learned the correct responses over the six blocks of the training phase (F[5,235] = 18.5, $p < .001$, $\eta^2_{partial}$ = 0.28), and performed better on the 80-20 vs 70-30 vs 60-40 pairs (F[2,94] = 32.2, $p < .001$, $\eta^2_{partial}$ = 0.41). In the subsequent test phase, performance was worse for novel pairs compared with those on which participants had trained (t[47] = 4.52, $p < .001$, d = 0.65), and we likewise saw performance declining over 80-20 vs 70-30 vs 60-40 trial types, $F(2,94) = 7.29$, $p = .001$, $\eta^2_{partial}$ = 0.13 (Figures S4B and S4C). Participants' behaviour therefore matched the same patterns seen in the original studies with this task (3,4).

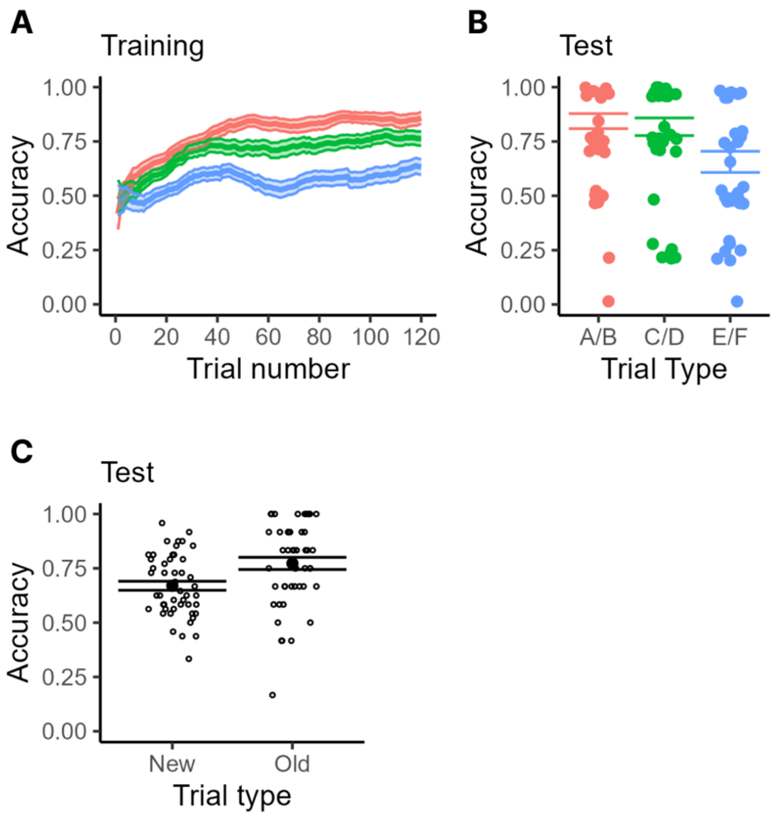

Figure S4. Performance on the Probabilistic Selection task during the training and test phases. A) Average performance over the course of the training, split by trial type. During training, participants gradually learned the correct responses, although performance was markedly better for the 80/20 (A/B) trial type compared with 70/30 (C/D) and 60/40 (E/F) trial types. B) Average accuracy during the test phase for just the trial types which had been trained previously. C) Average accuracy during the test phase for the trained vs. novel trial types. In B) and C) we show participants' individual data points, and the overall average$\pm$SE (error bars).

Full list of conditions and medications taken

Table S1. List of diagnosed conditions and medications taken by participants included in the final analysis (N=48).

**Diagnosis**	
Neurological disorder	0
Psychiatric disorder (ever diagnosed)	14
- Generalised or social anxiety disorder	9
- Major depressive disorder	3
- Other	2
Other chronic conditions	6
- Type 2 diabetes	1
- Inflammatory	2
- Other	3
**Medication**	
- Contraceptive pill	5
- Antidepressant (any)	4
- Antihypertensive	3
- Proton pump inhibitor	3
- Painkillers	2
- HRT	2
- Antispasmodic	1

Correlation between fasting glucose and iAUC

Fasting glucose was defined as the initial CGM reading taken prior to participants consuming the glucose drink (i.e., after the overnight fast). In our sample, fasting glucose and iAUC were modestly correlated ($r = 0.15$, $p = 0.30$). This correlation is rarely reported in previous studies, but in the available literature varies it varies from $r = 0.41$ to $r = 0.68$ (5,6).

Table S2. Comparison of the correlations between fasting glucose and iAUC, in the current and previous studies.

Study	Correlation between fasting glucose and iAUC *(r)*
Current study	0.15
Borg et al., 2010	0.68
Kjøllesdal et al., 2014	0.41

References

1. Betancourt, M. (2017). A conceptual introduction to Hamiltonian Monte Carlo. *ArXiv*. https://arxiv.org/abs/1701.02434
2. Vehtari et al. (2017). Practical Bayesian model evaluation using leave-one-out cross-validation and WAIC. *Statistics and Computing, 27,* 1413-1432.
3. Frank, M., Seeberger, L., O'Reilly, R. (2004) By carrot or by stick: Cognitive reinforcement learning in Parkinsonism. *Science*. 306, 1940–1943.
4. Frank, M., Moustafa, A., Haughey, H., Curran, T., & Hutchison, K. (2007). Genetic triple dissociation reveals multiple roles for dopamine in reinforcement learning. *Proc. Natl. Acad. Sci. USA,* 104, 16311–16316.
5. Kjøllesdal, M. S., Øverby, N. C., & Andersen, L. F. (2014). Blood glucose measured at several time points and correlation with incremental area under the curve. *Journal of Nutrition & Food Sciences*, 4(6), 1000315. https://doi.org/10.4172/2155-9600.1000315
6. Borg, R., Kuenen, J. C., Carstensen, B., Zheng, H., Nathan, D. M., Heine, R. J., Nerup, J., Borch-Johnsen, K., & Witte, D., R. (on behalf of the ADAG Study Group). (2010). Associations Between Features of Glucose Exposure and A1C: The A1C-Derived

Average Glucose (ADAG) Study. *Diabetes*, *59* (7), 1585–
1590. https://doi.org/10.2337/db09-1774
